# Comparison of a Presbyopia-Correcting Supplementary Intraocular Lens Combination and a Capsular-Bag Lens: An In Vitro Study

**DOI:** 10.3390/diagnostics13081482

**Published:** 2023-04-20

**Authors:** Ramin Khoramnia, Isabella Diana Baur, Weijia Yan, Grzegorz Łabuz, Gerd Uwe Auffarth

**Affiliations:** David J. Apple Center for Vision Research, Department of Ophthalmology, University of Heidelberg, Im Neuenheimer Feld 400, 69120 Heidelberg, Germany

**Keywords:** polypseudophakia, trifocality, optical quality

## Abstract

We evaluated the optical quality of two approaches to trifocality: polypseudophakia versus monopseudophakia. The combination (polypseudophakia) of a monofocal Basis Z B1AWY0 and AddOn Trifocal A4DW0M intraocular lens (IOL) was compared to using one Basis Z Trifocal B1EWYN IOL, all from 1stQ GmbH. In both approaches, we measured modulation transfer function (MTF) and Strehl Ratio (SR) values at 3.0 and 4.5 mm pupil sizes. We determined the through-focus (TF) MTF at 25, 50 and 100 lp/mm for the 3 mm aperture. United States Air Force (USAF) target images were recorded. MTF measurement of the trifocal lens and the combined monofocal and trifocal AddOn IOL showed good performance at the far and near focus for the 3 mm aperture. For the 4.5 mm aperture the MTF improved for the far focus but decreased for the intermediate and near focus. TF MTF showed better contrast at the far focus for the polypseudophakic setup but at the expense of the efficiency at the near focus. However, the USAF chart images revealed only minimal differences between both approaches. The optical quality of the polypseudophakic approach was not affected by the presence of two IOLs instead of one and proved to be comparable with the performance of one capsular-bag-fixated trifocal IOL. Differences between the single vs. two-lens approach seen in the TF MTF analysis could be attributed to the optical design that varied between the trifocal models.

## 1. Introduction

Patients who undergo cataract surgery can nowadays opt for different presbyopia-correcting intraocular lenses (IOLs). Trifocal lenses create foci for far, intermediate and near distance to reduce the need for spectacles at these distances [1]. Although many patients can benefit from trifocal IOLs, the more frequently implanted are monofocal IOLs [2].

The development of supplementary IOLs which have trifocal optics, designed for implantation into the ciliary sulcus, opened the possibility for monofocal pseudophakic patients to gain trifocality with this second lens. Another possible approach is the combined implantation in one surgery session of a monofocal or monofocal-toric IOL into the capsular bag followed by implantation of a supplementary trifocal IOL into the ciliary sulcus. In patients who may develop ocular pathologies during the course of their lives that lead to a loss of function, a trifocal IOL may become disadvantageous and the only treatment option is its removal. The combined implantation allows one to achieve trifocality that is more easily reversible compared to removing a capsular bag-fixated trifocal IOL [3].

IOLs that are intended for sulcus fixation are designed to prevent contact with the capsular bag fixated IOL and iris tissue and associated complications like interlenticular opacification, hyperopic shift, pigment dispersion and elevated intraocular pressure [4,5,6,7]. A sulcus-fixated supplementary IOL can be removed from the eye more easily than a capsular bag fixated IOL. The reversibility of this surgical presbyopia correction seems beneficial in a variety of situations, namely in patients with an elevated risk of deviation from the target refraction or in the case of a future loss of function due to an ocular pathology [8,9,10,11].

The secondary implantation of the supplementary AddOn Trifocal A4DW0M (1stQ GmbH, Mannheim, Germany) has been studied in several clinical trials. They found that the implantation of the secondary IOL in pseudophakic eyes yielded very good results in terms of spectacle independence which are comparable to those of capsular-bag fixated trifocal IOLs [12,13]. This was also demonstrated for a patient collective with previous laser vision correction [8]. A recently published retrospective clinical study by Harrisberg et al. compared primary duet procedure, the combined implantation of a monofocal or monofocal-toric IOL in the capsular bag and of the multifocal version of the 1stQ AddOn IOL into the ciliary sulcus during the same surgery, to the implantation of a single multifocal IOL with a design comparable to that of the additive IOL into the capsular bag. They did not find statistically significant differences in uncorrected distance and near visual acuity or postoperative spherical equivalent refraction [14]. Clinical data suggest that the primary duet procedure using the 1stQ AddOn IOL might be a valuable option when refining IOL selection for a patient asking for presbyopia correction.

The possibility of creating trifocality using two intraocular lenses has raised the question if the polypseudophakic approach can offer similar image quality compared to having one trifocal IOL that is implanted in the capsular bag. In this in vitro study, we assessed the optical quality in both approaches.

## 2. Materials and Methods

### 2.1. Intraocular Lenses

We assessed three IOL models from one manufacturer, (1stQ GmbH, Mannheim, Germany) measuring two samples of each model:

- monofocal Basis Z B1AWY0 with a dioptric power of +20.0;
- Basis Z Trifocal B1EWYN with a dioptric power of +20.0 and near addition of +3.5 diopters for capsular bag fixation;
- AddOn Trifocal A4DW0M with a dioptric power of 0.0 and near addition of +3.0 diopters for sulcus implantation.

All IOLs studied share the same refractive index of 1.46. The monofocal Basis Z and trifocal Basis Z share the same one-piece platform made of hydrophilic-acrylate material with a 25% water content and a hydrophobic surface. The trifocal AddOn is also made of hydrophilic-acrylate with a 25% water content and features a square design with four flex haptics to prevent iris capture. Both capsular bag fixated IOLs feature an aspheric design, whereas the AddOn IOL features a spherical design.

**Figure 1:**
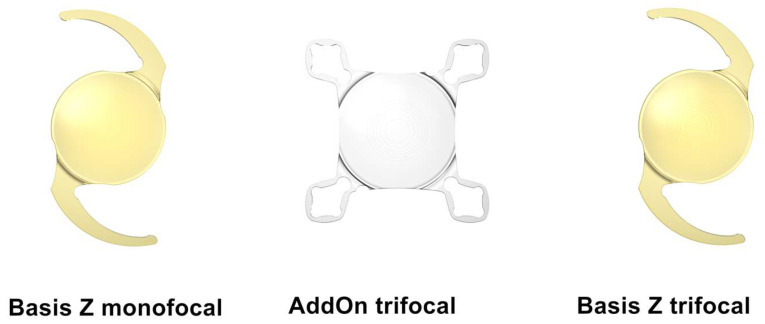
The three IOL models studied. The Basis Z and Basis Z trifocal share the same platform with C-loop haptics and are implanted in the capsular bag. The AddOn trifocal features a square design with four haptics and is implanted into the ciliary sulcus.

Both trifocal lenses have diffractive optics; however, the designs of the lenses are not identical. The AddOn IOL has 6 diffractive rings, while the capsular lens has 7 diffractive rings. Figure 1 shows the three IOL models included in this analysis.

### 2.2. Optical Metrology

We used the OptiSpheric IOL PRO2 (Trioptics GmbH, Wedel, Germany) to assess the optical performance of both the single trifocal IOL and the combined AddOn and monofocal IOLs. This setup complies with the ISO standard for optical properties and test methods [15]. Different targets, a crosshair or a USAF target image, can be selected. A light source illuminates the target after passing a collimator and the IOL under test focuses the selected target. A microscope objective and a charged-coupled device (CCD) camera capture the projected image. The IOL(s) under test were submerged in balanced salt solution in the IOL holder. The line spread function was derived from the projected image of the test target and the modulation transfer function (MTF) was calculated using Fourier Transformation.

The MTF measurements were performed using an aberration-neutral cornea model. We used monochromatic light with a wavelength of 546nm for this measurement at a 3.0 mm and 4.5 mm aperture.

The CCD camera was then moved along the focal planes and the through-focus (TF) MTF at a set spatial frequency (25, 50 and 100 lp/mm) was captured to assess the through-focus MTF. The defocus ranged from +1.0 to −4.5 diopters, starting from the best far focus.

The Strehl ratio was calculated by weighting the area under the MTF curve by the diffraction-limited MTF curve [16,17]. This ratio is helpful in evaluating overall optical quality, where a lower Strehl ratio indicates a poorer optical quality.

Using a different target, a US Airforce resolution chart, photographs were taken at the best far, intermediate and near focus. These images were used to qualitatively assess and compare the image quality of the different IOLs.

To simulate polypseudophakia we placed two IOLs in a holder, which resulted in a 2 mm separation between the monofocal and the AddOn IOL. In vivo, the sulcus fixated and capsular bag fixated IOL are in closer proximity with a distance of only 450 μm [18]. In the human eye, the lens position has a considerable effect on the refractive error [19]. In vitro, however, this is compensated by the adjustable position of the CCD camera [20].

All measurements were performed three times and the results from the two samples were averaged. MTF data were analyzed using routines written in MATLAB (MathWorks, Portola Valley, CA, USA). The wide MTF value between 0 and 1 and the low standard deviation of optical quality measurements, typically between 0 and 0.02 [21], eliminate the need to apply statistical difference tests between IOLs.

## 3. Results

**Figure 2:**
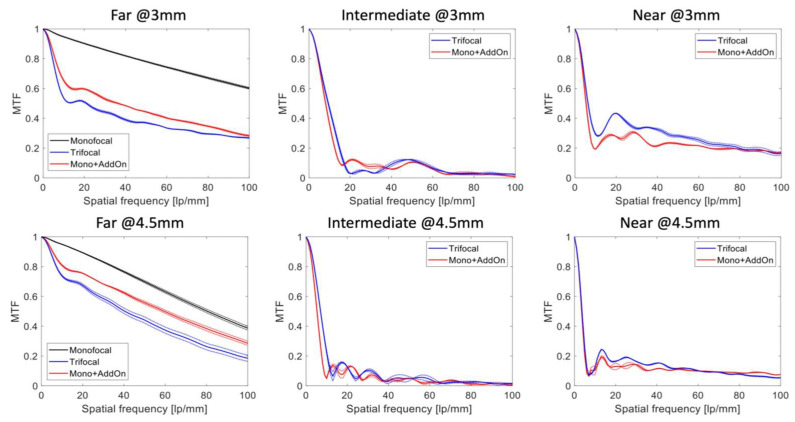
The modulation transfer function (MTF) of the monofocal, and single-lens trifocal and Mono-AddOn measured at a 3 and 4.5 mm pupil. The dotted lines show the values of each lens separately, the solid lines refer to the average of two IOLs.

Figure 2 shows the average MTF curves of the studied IOLs measured at the best far, intermediate and near focus. The position of the near-point depended on the add power, which for the combined monofocal and AddOn (Mono + AddOn) was 2.94 ± 0.03 D, and for the trifocal IOL it was 3.30 ± 0.02 D. The intermediate focus was detected at approx. 1.40 D (single lens) and 1.76 D (two IOLs) using the TF MTF at 50 lp/mm.

**Figure 3:**
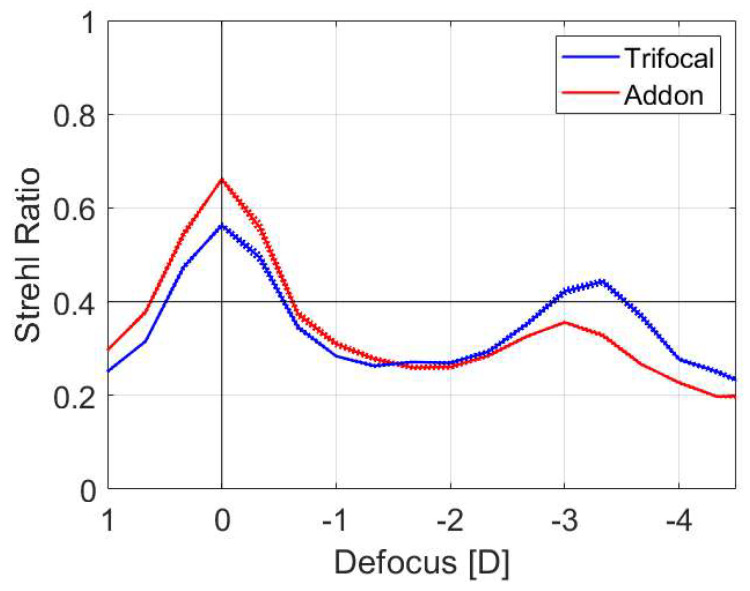
Strehl Ratio as a function of defocus. A higher Strehl Ratio indicates a better optical quality. Both the trifocal and AddOn IOL showed two peaks, corresponding to the far and near focus.

Table 1 summarizes the Strehl ratio results measured at the far, intermediate and near position for the 3 mm and 4.5 mm apertures. Figure 3 shows the Strehl Ratio as a function of defocus.

**Figure 4:**
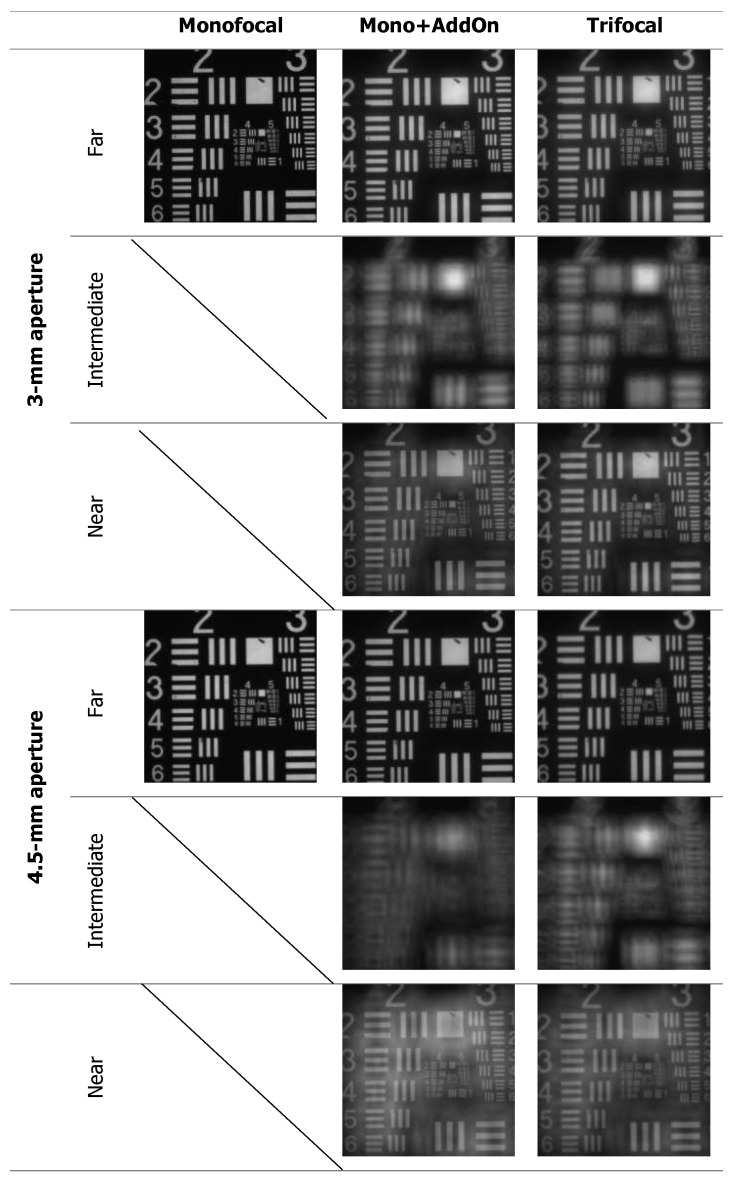
USAF target images recorded at the far, intermediate and near focus and apertures 3 mm and 4.5 mm.

Figure 4 shows the USAF resolution chart photographs, which were taken at the best-focus position.

For both apertures, the monofocal lens outperformed the other lenses at far-point (Figure 1 and Figure 2, Table 1), but at 4.5 mm this disparity became less distinct.

The Mono + AddOn produced higher MTF levels at the far focus compared to the single trifocal IOL. For the near focus, however, the trifocal showed slightly higher MTF values at the 3 mm aperture (Figure 1). At the intermediate focus both setups showed close MTF values.

At 4.5 mm, the MTF levels at the intermediate and near focus were comparable for the Mono + AddOn and trifocal lenses (Table 1, Figure 1). In the USAF target images, only small differences for the near and intermediate focus in favor of the trifocal IOL are noticeable (Figure 2), which is in agreement with Figure 1.

**Figure 5:**
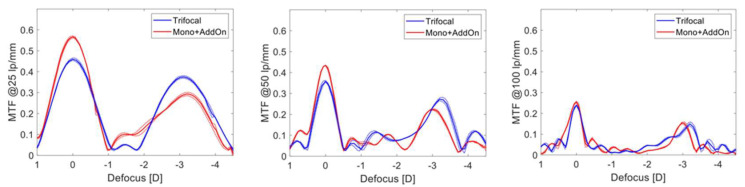
The through-focus modulation transfer function (MTF) of the single-lens trifocal and Mono + AddOn IOLs at 25, 50 and 100 lp/mm for the 3 mm aperture.

The results of the TF MTF measurements at a frequency of 25, 50 and 100 lp/mm for the 3 mm pupil are shown in Figure 5. The analysis demonstrated that the Mono + AddOn have a larger allocation of energy to the far focus compared to the trifocal IOL, which in contrast showed a higher peak for the near focus at 25 and 50 lp/mm. Both setups revealed a clear separation of TF MTF peaks corresponding to the far and near main foci. The intermediate focus was less delineable.

The difference in the near-focus position resulted from different add powers of the studied lens models. The TF MTF of the IOLs was close between those of the trifocal IOL and Mono + AddOn with minimally better near performance of the former.

## 4. Discussion

Both the Mono + AddOn setup and the Basis Z Trifocal B1EWYN IOL provided good MTF performance at the far and near focus for the 3 mm aperture. At the 4.5 mm aperture, however, image quality improved for the far focus at the expense of the near focus. The optical performance of the combination of the monofocal and AddOn IOL was not inferior to that of the capsular-bag trifocal Basis Z.

The near-focus MTF of the standard trifocal lens was minimally better, which we attribute to differences in the design since the capsular trifocal IOL has seven diffractive rings, while the AddOn has six diffractive rings. The add power also differs with 3.5 D for the standard trifocal and 3.0 D for the AddOn IOL, which is reflected in the different positions of the secondary peaks in the through-focus scan (Figure 3). The USAF chart images showed only minimal differences, which confirms that similar image quality is achieved with both approaches.

The results we obtained in this study are in good agreement with a laboratory study we performed using a different supplementary IOL, the Sulcoflex (Rayner Intraocular Lenses Ltd., Worthing, West Sussex, UK) [20]. The combination of the trifocal supplementary Sulcoflex IOL and the RayOne aspheric (Rayner Intraocular Lenses Ltd.) provided comparable optical quality to the RayOne Trifocal (Rayner Intraocular Lenses Ltd.), with very similar MTF curves. The US Air Force target images of both the polypseudophakic and the standard trifocal approach were indistinguishable for far, near and intermediate focus [20]. We found analogous results for the 1stQ lenses.

Furthermore, the study of the Rayner lenses assessed light attenuation with both approaches, as the implantation of two IOLs creates four interfaces instead of two, as would be the case with only one IOL. The calculated reflectance for the Sulcoflex and RayOne IOLs was 0.8% [20]. As it depends on the difference in the refractive indices of the materials forming the interface, the reflectance can be expected to be higher with an IOL with a higher refractive index [22,23]. The 1stQ trifocal AddOn and Basis Z share the same refractive index of 1.46 with the Sulcoflex and RayOne aspheric IOLs. Therefore, the theoretical reflectance is the same for both IOL systems and even lower than the values calculated for certain capsular bag IOLs with a higher refractive index [20]. We conclude that the implantation of the two 1stQ IOLs we studied does not increase the reflectance to a level that could negatively affect image quality.

It had been previously reported that the IOL’s surface geometry is of greater importance than the light reflection from several interfaces [23,24]. Schrecker et al. compared the reflectance of the monofocal MC 5812 AS to the combination of the same IOL and the additive MS 714 PB (both manufactured by Dr. Schmidt Intraocularlinsen GmbH, St. Augustin, Germany). They calculated the signal-to-noise ratio (SNR) and they found no negative effects on SNR due to the presence of two IOLs [23].

The reflected light spreads over a wider area of the retina than does light that is focused by the IOL(s). The SNR is a measure that describes the ratio of the desired signal, in this case the light transmitted by the IOL(s), and the background noise, in this case the reflected light or glare. A decreased SNR could, therefore, indicate an increased level of glare [23]. In the study by Łabuz et al., the Weber fraction was determined to examine the effect of the light loss measured on the visual perception [20]. Weber’s law describes the increment needed to detect a difference between two stimuli, and among other areas of human perception it also applies to visual function. As the Weber fraction calculated from the differences in optical power between a polypseudophakic and one-IOL approach was well below the level of the Weber fraction determined in healthy subjects observing a white light stimulus [25], it is unlikely that such small differences could be noticeable to the human eye [20].

In the previously mentioned study by Łabuz et al. [20], the light transmission was not only calculated but also measured. Optical power measurements revealed a mean light loss of 1.3% with the polypseudophakic approach compared to the single IOL. This effect was considered to have negligible clinical impact [20].

MTF values for different trifocal IOLs have been published in the literature. Carson et al. reported the MTF values at 50 lp/mm of 0.402, 0.153, and 0.181 at the far, intermediate and near focus with a 3 mm aperture for the PanOptix. For the AT Lisa Tri, it was 0.388, 0.118 and 0.199, and for the FineVision Micro F, it was 0.361, 0.096 and 0.165 for far, intermediate and near with the same pupil size [26]. When compared with the MTF levels found in the current study, single-lens trifocal and Mono + Add appear comparable or better at far (MTF = 0.370 and MTF = 0.443, respectively), and near (MTF = 0.281 and MTF = 0.223, respectively). However, this single-frequency parameter is worse at the intermediate range, with the single and two-IOL approaches showing lower MTF values at the intermediate range (MTF = 0.121 and MTF = 0.106, respectively) than the PanOptix, but close to that of the AT Lisa Tri and higher than the FineVision Micro F. Although the comparison of the subjective image quality indicates a better overall (multi-frequency) optical quality of the established trifocals at the intermediate range, the Basis Z and AddOn models demonstrated advantages at far and near points. Similar conclusions can be drawn from the comparison of the USAF resolution test images of the Sulcoflex Trifocal and the RayOne Trifocal measured in polychromatic light [17] with the (monochromatic) images presented in Figure 2 of the current study. The clinical impact of the observed differences warrants further research.

The effect of misalignment of the Sulcoflex IOL was evaluated as well [20]. A decentration of 0.2, 0.4 and 0.6 mm as well as 1.8 mm were examined. The decentration of up to 0.4 mm did not affect image quality at the different foci, while a decentration of 0.6 mm lead to a minimal deterioration of the optical quality at the intermediate focus. For a severe decentration of 1.8 mm, an increased MTF value at the far focus was observed, while image quality decreased considerably for the intermediate and near focus. Tilting the supplementary IOL by 5° did not affect optical quality. Overall the study revealed a high tolerance to tilt and moderate decentration of the supplementary IOL [20].

In a clinical study by Palomino-Bautista et al., no decentration or tilt was seen with the 1stQ trifocal AddOn [12]. Valvecchia et al. used ultrasound biomicroscopy for follow-up of patients who underwent secondary implantation of the trifocal version of the 1stQ AddOn IOL and did not observe tilt in their patient collective [27]. Although clinical data show very good centration and sufficiently predictable position of the 1stQ AddOn IOL, in analogy to the assessment of decentration of the Sulcoflex, a moderate decentration or tilt should not significantly affect the optical quality. The 1stQ AddOn we assessed in this study is also available in a toric version. For the toric IOL model, not only tilt and decentration are of importance, but rotational stability is crucial to achieve good refractive outcomes. Gundersen et al. in a clinical study, found rotational stability for the 1stQ AddOn toric that was comparable to that of a capsular-bag fixated toric IOL with a mean rotation of less than 5 degrees [28].

Data published for the Sulcoflex IOL suggest inferior rotational stability, which may be related to the C-loop design of this IOL model. McLintock et al. found a mean rotation of the Sulcoflex toric of 8.2 degrees on the first postoperative day and they had to reposition 62% of the supplementary IOLs during the follow-up period of three months [29]. Meyer et al. observed several cases of postoperative IOL rotation in keratoconus patients with the toric Sulcoflex IOL [30]. By contrast, Gundersen et al. reported that no repositioning of the supplementary toric 1stQ IOL was necessary in their study including 18 eyes [28]. The four loop-haptics of the 1stQ AddOn seem to contribute to rotational stability and good centration of the IOL.

Supplementary sulcus-fixated IOLs are used in various indications. The monofocal and monofocal-toric versions of the 1stQ AddOn have been successfully used to correct residual refractive errors in pseudophakic patients including patients with a history of penetrating keratoplasty [28,29,31,32]. The trifocal version of this IOL can be an option for pseudophakic patients who wish to be more independent from spectacles as it provides in vivo results similar to a capsular-bag fixated multifocal IOL [8,12,13].

Another indication for the use of supplementary IOLs is the combined implantation of a monofocal IOL into the capsular bag and of a trifocal IOL into the ciliary sulcus during the same surgical procedure. This technique was described using the Sulcoflex IOL and provided comparable results to those reported for capsular-bag fixated trifocal IOLs while providing the advantage of an exit strategy due to its reversibility [9,10,11]. The clinical data on the combined implantation of a monofocal capsular bag-fixated IOL and a trifocal sulcus-fixated IOL are supported by our laboratory evaluation, as we found comparable results for the combination of the trifocal AddOn and monofocal IOL and the trifocal IOL in our laboratory evaluation.

Our study supports the available data that the polypseudophakic approach has no disadvantage over the classical approach with a trifocal IOL in the capsular bag related to optical quality. However, in certain situations, the polypseudophakic approach may have advantages because it is easier to reverse. A retrospective clinical study by Harrisberg et al. showed that the safety profile and outcomes are equivalent for both the primary duet procedure and the implantation of a single multifocal IOL. Visual outcomes, complication rates and intraocular pressure did not differ in both treatment groups [14]. The AddOn IOL can also be used in already pseudophakic eyes after previous monofocal IOL implantation into the capsular bag to offer patients the possibility of presbyopia correction even years after cataract surgery. In addition, the polypseudophakic approach appears to be robust against misalignment [20,33]. We therefore consider this procedure as a useful option for presbyopia treatment.

A limitation of our study is that the near addition for the single trifocal IOL is +3.0 D, while the addition for the combination Mono + Add is +3.5 D, which makes direct comparison difficult. However, it is common practice to compare IOLs with different near additions in terms of optical quality [34,35,36,37,38].

In conclusion, we found that the combination of a monofocal IOL and trifocal AddOn IOL did not decrease the optical quality when compared to one capsular bag-fixated trifocal IOL. The polypseudophakic approach may be advantageous in several indications due to its reversibility.

## Figures and Tables

**Table 1:** Strehl ratio values of the analyzed lenses measured at the best far, near and intermediate focus.

Aperture	Focus	Monofocal	Mono-AddOn	Trifocal
3 mm	Far	0.97 ± 0.00	0.65 ± 0.01	0.56 ± 0.01
	Intermediate		0.26 ± 0.00	0.27 ± 0.01
	Near		0.35 ± 0.00	0.44 ± 0.00
4.5 mm	Far	0.91 ± 0.00	0.78 ± 0.00	0.69 ± 0.02
	Intermediate		0.18 ± 0.00	0.22 ± 0.01
	Near		0.20 ± 0.00	0.23 ± 0.00

## Data Availability

The datasets used and/or analysed during the current study are available from the corresponding author on reasonable request.
